# Coumarin-Based Hybrids: From Linear Photophysical Properties to Multiphoton Excitation Behavior

**DOI:** 10.1021/acsphyschemau.6c00010

**Published:** 2026-05-14

**Authors:** Luis M. G. Abegão, Leonardo R de Almeida, Juliana G. M. Lima, Luciana M. Ramos, Hamilton B. Napolitano, João V. Valverde, Cleber R. Mendonça, Leonardo De Boni, Leandro H. Z. Cocca

**Affiliations:** † Sensors, Instrumentation and Applied Photonics Group, Department of Physics, Federal University of Sergipe, São Cristóvão 49107-230, Brazil; ‡ Laboratory of Medicinal Chemistry and Organic Synthesis, State University of Goiás, Anápolis 76600-000, Brazil; § Photonics Group, Institute of Physics of São Carlos, University of São Paulo, São Carlos 13563-120, Brazil; ∥ Photonics Group, Institute of Physics, 67824 Federal University of Goiás, Goiânia 74690-900, Brazil

**Keywords:** benzothiazole, benzimidazole, photophysical properties, two-photon excitation, three-photon excitation, photoluminescent probes

## Abstract

Coumarin-based derivatives are recognized as tunable photonic building blocks due to their strong light–matter interaction and relevance for both linear and nonlinear optical applications. This work presents an investigation of the linear optical properties and multiphoton excitation response of four derivatives coupled with benzothiazole and benzimidazole moieties. The compounds 7-diethylamino-coumarin-benzothiazole, 6-bromo-coumarin-benzothiazole, and 7-diethylamino-coumarin-benzimidazole exhibit nearly identical absorption maxima (∼423–428 nm) and emission peaks (∼485 nm), while displaying pronounced differences in molar absorptivity and ground to first excited-state transition dipole moment (μ_01_). The 7-diethylamino-substituted derivatives show enhanced molar absorptivity (∼5.3 × 10^4^ L mol^–1^ cm^–1^) and larger transition dipole moment (μ_01_ ∼ 8.1 D) compared to the bromo-substituted (μ_01_ ∼ 5.5 D). In contrast, 7-hydroxyl-coumarin-methyl-benzimidazole exhibits a distinct spectral signature characterized by a larger Stokes shift and lower molar absorptivity, with an intermediate μ_01_ ∼ 7.0 D reflecting a different electronic balance within the conjugated framework. Multiphoton excitation experiments using femtosecond laser pulses demonstrate efficient two-photon (800 nm) and three-photon (1200 nm) excited fluorescence for all derivatives. Remarkably, the hydroxylated derivative combines an exceptionally high fluorescence quantum yield (∼48%) with a measurable excited-state lifetime (∼3 ns), identifying it as the most promising candidate for bright photoluminescent probing under one-, two-, and three-photon excitation.

## Graphical abstract

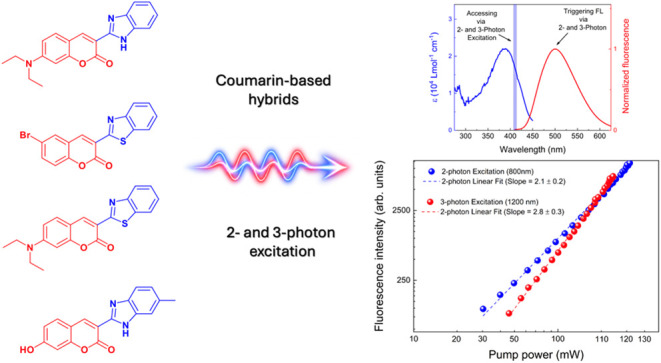

## Introduction

Coumarin derivatives are a prominent class of heterocyclic compounds known for their diverse biological activities and chemical reactivity.^1,2^ The benzopyrone core structure, consisting of a benzene ring fused to a lactone ring, is a versatile scaffold in medicinal chemistry.^3–5^ This core has been extensively modified in numerous derivatives,^6^ including the four compounds investigated in this work: 3-(benzo­[*d*]­thiazol-2-yl)-7-(diethylamino)-2*H*-chromene-2-one (CAA-31k), 3-(benzo­[*d*]­thiazol-2-yl)-6-bromo-2*H*-chromen-2-one (CBA-31i), 3-(1*H*-benzo­[*d*]­imidazol-2-yl)-7-(diethylamino)-2*H*-chromene-2-one (CAF-31j), and 7-hydroxy-3-(6-methyl-1*H*-benzo­[*d*]­imidazol-2-yl)-2*H*-chromen-2-one (CHMF-31f).^7^ Such derivatives have gained significant attention for their applications as therapeutic agents,^8,9^ including anticoagulants,^10^ antimicrobial agents,^11–13^ and anticancer.^14,15^ Coumarins are also valued for their optical properties,^16–19^ making them useful in photoluminescence-based applications and organic electronics.^20,21^

In addition, coumarin derivatives have emerged as highly versatile functional molecules in advanced photochemical and photonic technologies.^22,23^ Owing to their tunable electronic structures, high molar absorptivities, and efficient radiative and nonradiative deactivation pathways, these compounds have been extensively explored as fluorescent probes and chemosensors for the selective detection of metal ions, reactive species, and environmental analytes.^24^ In parallel, their favorable excited-state redox properties and strong light–matter interaction have enabled their use as efficient photoinitiators and photoredox catalysts in free-radical and cationic polymerization processes, including conjugated polymer synthesis under visible and near-infrared irradiation.^25^ Particularly, coumarin-based architecture has also shown outstanding performance as nonlinear photoinitiators for additive manufacturing, enabling two-photon polymerization with subwavelength spatial resolution.^26^ In this way, these studies highlight coumarin derivatives as a multifunctional molecular platform at the interface of chemistry, materials science, and photonics, with broad implications for sensing, imaging, and light-driven technologies. In this context, this study presents a comprehensive investigation of the linear optical properties as well as the two- and three-photon excitation behavior of four coumarin derivatives–CBA-31i, CAA-31k, CAF-31j, and CHMF-31f–all based on a coumarin chromophoric core. In CBA-31i and CAA-31k, the coumarin unit is π-conjugated to a benzothiazole-based acceptor, whereas in CAF-31j and CHMF-31f it is linked to a benzimidazole moiety. In all compounds, the donor coumarin fragment and the coupled nuclei acceptor are connected through a π-conjugated C=C linker, giving rise to a push–pull Donor-π-bridge-Acceptor (D−π–A) molecular architecture that is favorable for charge-transfer-mediated optical processes. Details regarding the synthesis of all investigated compounds can be found elsewhere.^7^

In CBA-31i, the introduction of an electron-withdrawing bromine substituent enhances the electrophilicity and redox activity of the coumarin scaffold, increasing its chemical reactivity and potential for biological interactions, as reported for related fluorescent 3-heteroarylcoumarin inhibitors of anaplastic lymphoma kinase.^27^ In contrast, CAA-31k features a diethylamino substituent within the coumarin framework, which modulates lipophilicity and membrane permeability, potentially improving bioavailability.^28^ In both compounds, the presence of a thiazole ring further broadens their biological and photonic relevance, as thiazole-containing coumarins are known to engage in strong π–π and hydrogen-bonding interactions, making them attractive candidates for medicinal chemistry and biophotonic applications, particularly as photoluminescent bioprobes.^29,30^

In CAF-31j, the diethylamino group acts as a strong electron donor, promoting intramolecular charge transfer and enhancing optical absorption, emission, and photoluminescence efficiency, which are desirable for fluorescence-based bioimaging and sensing.^31,32^ Conversely, CHMF-31f contains a hydroxyl substituent that introduces hydrogen-bonding capability and modulates excited-state dynamics, favoring environmental sensitivity and biological compatibility.^33–35^ Both compounds incorporate a benzimidazole moiety, which strengthens π-conjugation, charge-transfer interactions, and photostability. The resulting D−π–A architectures highlight their potential for applications in medicinal chemistry, biophotonics, and nonlinear optics as optically active functional materials.^36,37^

Our thorough investigation employed ultraviolet–visible absorbance and fluorescence spectroscopy to assess the solvatochromic behavior of these compounds, offering an understanding of how solvent polarity modulates their electronic transitions. Additionally, fluorescence lifetime decay and quantum yield was measured to better understand the photoluminescent properties of the investigated compounds.

Beyond their linear optical properties, the potential of all investigated compounds for multiphoton excitation was evaluated using ultrashort laser pulses, revealing efficient fluorescence under both two- and three-photon excitation. This behavior significantly broadens their applicability in advanced imaging techniques, particularly in multiphoton microscopy for deep-tissue bioimaging.^38^ The comprehensive set of optical parameters investigated—including transition dipole moment, Stokes shift, photoluminescence quantum yield, and multiphoton excitation potential—offers a robust foundation for applications in organic light-emitting diodes (OLEDs),^39^ fluorescent sensors,^29^ and biomedical imaging.^40^ By integrating these optical characterizations with structural properties, this study advances our understanding of the relationship between molecular structure and photophysical behavior, supporting the exploration of coumarin-derivatives compounds as potential photonic devices or emissive bioprobes.

## 2 Experimental Section

### 2.1 Investigated Compounds

The molecular structure of the coumarin-based hybrid derivatives in this study is presented in Figure 1. Lima and co-workers^7^ previously reported its synthesis, introducing a green, ionic-liquid-mediated synthetic route for coumarin-based hybrids. Using methyl-acetyl-imidazole chloride (MAI·Cl) as a Brønsted-acidic ionic liquid catalyst^41,42^ (40 mol % loading) in ethanol at 80 °C to avoid hazardous reagents such as POCl_3_ and minimize waste generation. The catalyst is recyclable, nonvolatile and compatible with aqueous workup, aligning with principles 5 (safer solvents and auxiliaries), 6 (design for energy efficiency), 7 (use of renewable feedstocks) and 9 (catalysis) of green chemistry.^43^ Beyond the catalytic system, the study incorporated multimodal characterization, including FTIR, ^1^H and ^13^C­{^1^H} NMR, DFT-based quantum-chemical descriptors and principal component analysis (PCA) of molecular properties, to rationalize structure–activity relationships for antibacterial and antioxidant assays. All multimodal characterization data and analysis are provided here.^7^

**Figure 1:**
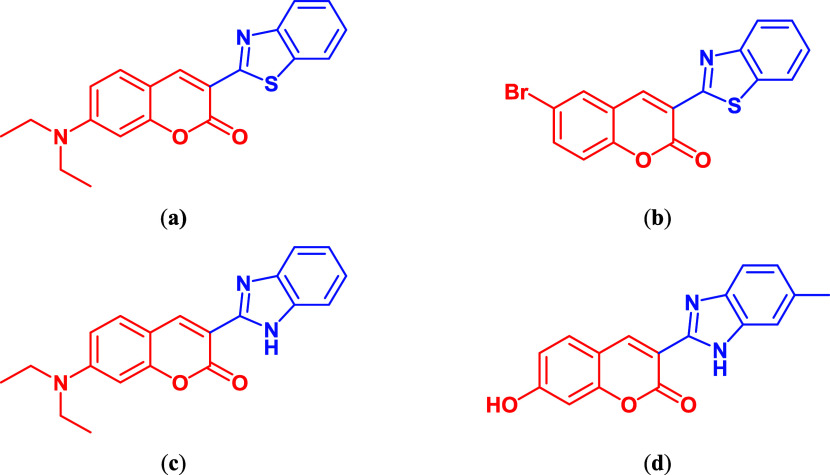
Molecular structures of four coumarin-based hybrid derivatives (a) CAA-31k, (b) CBA-31i, (c) CAF-31j, and (d) CHMF-31f investigated in this work.

### 2.2 Optical Properties

The coumarin derivatives investigated in this work were dissolved in dimethyl sulfoxide (DMSO) at a concentration of approximately 10^–5^ mol L^–1^ for UV–vis absorbance measurements, which were performed using a spectrophotometer (Shimadzu, UV-1800, Japan) in 10 mm path-length fused quartz cuvettes. Emission spectra and fluorescence anisotropy were recorded at room temperature with samples at a concentration of about 10^–6^ mol L^–1^, using the same cuvettes, on a fluorescence spectrophotometer (Hitachi, F7000, Japan). All measurements were repeated five times, with no evidence of aggregation or photodegradation.

Fluorescence lifetime and multiphoton excitation experiments were performed using a femtosecond laser system (Pharos, Light Conversion, Lithuania) coupled to an optical parametric amplifier (Orpheus, Light Conversion, Lithuania). The laser operated at a repetition rate of 1 kHz, delivering pulses with a duration of approximately 200 fs. Lifetime decay measurements were carried out by exciting the samples at their absorption maxima. The fluorescence signal was collected using an optical fiber positioned at 90° with respect to the excitation beam in order to minimize scattered light. The collected emission was guided through the optical fiber to a high-speed photodiode detector (Precision Applied Science, PD1000, Slovakia) with a temporal resolution of approximately 700 ps. The photodiode output signal was recorded using a 1 GHz digital oscilloscope (Tektronix, 7104C, USA), allowing the temporal profile of the fluorescence decay to be monitored.

For multiphoton excitation experiments, the compounds were excited at 800 nm for two-photon absorption and 1200 nm for three-photon absorption. Fluorescence emission spectra were collected within a controlled black box environment to eliminate interference from external light, using a photomultiplier tube (Hamamatsu, H5783P, Japan). A set of optical filters was used in both the excitation and emission paths to optimize signal detection. In the excitation path, an infrared blocking filter (TF1, Thorlabs, USA) was employed to prevent stray infrared light. In the emission path, a bandpass filter (FBH05488–10, Thorlabs, USA) was used to isolate fluorescence emission around the primary fluorescence peak. Fluorescence was collected at 90 deg relative to the excitation beam path. The fluorescence intensity was recorded as a function of laser power, and log–log plots of intensity versus power were used to confirm multiphoton absorption behavior.

## 3 Results and Discussion

### 3.1 Absorption, Emission, and Transition Dipole Moment

Figure 2 illustrates the absorption and emission spectra for the four coumarin derivatives CAA-31k, CBA-31i, CAF-31j, and CHMF-31f in DMSO. The comparative analysis of CAA-31k, CBA-31i, and CAF-31j highlights that these three molecules exhibit nearly identical maximum absorption wavelengths, with only a 5 nm difference (423 nm for CAA-31k and 428 nm for CBA-31i and CAF-31j), as well as the same emission peak at *c.a*. 485 nm. However, a key distinction lies in their molar absorptivity (ε), with CAA-31k and CAF-31j showing a higher ε *c.a.* 5.3 × 10^4^ L mol^–1^ cm^–1^ compared to CBA-31i (ε ∼ 2.4 × 10^4^ L mol^–1^ cm^–1^). In contrast, CHMF-31f exhibits a markedly different spectral response, with a blue-shifted absorption maximum at *c.a*. 388 nm and a red-shifted emission centered at *c.a.* 500 nm, resulting in a substantially larger Stokes shift. CHMF-31f displays the lowest ε ∼ 2.2 × 10^4^ L mol^–1^ cm^–1^ of all investigated samples.

**Figure 2:**
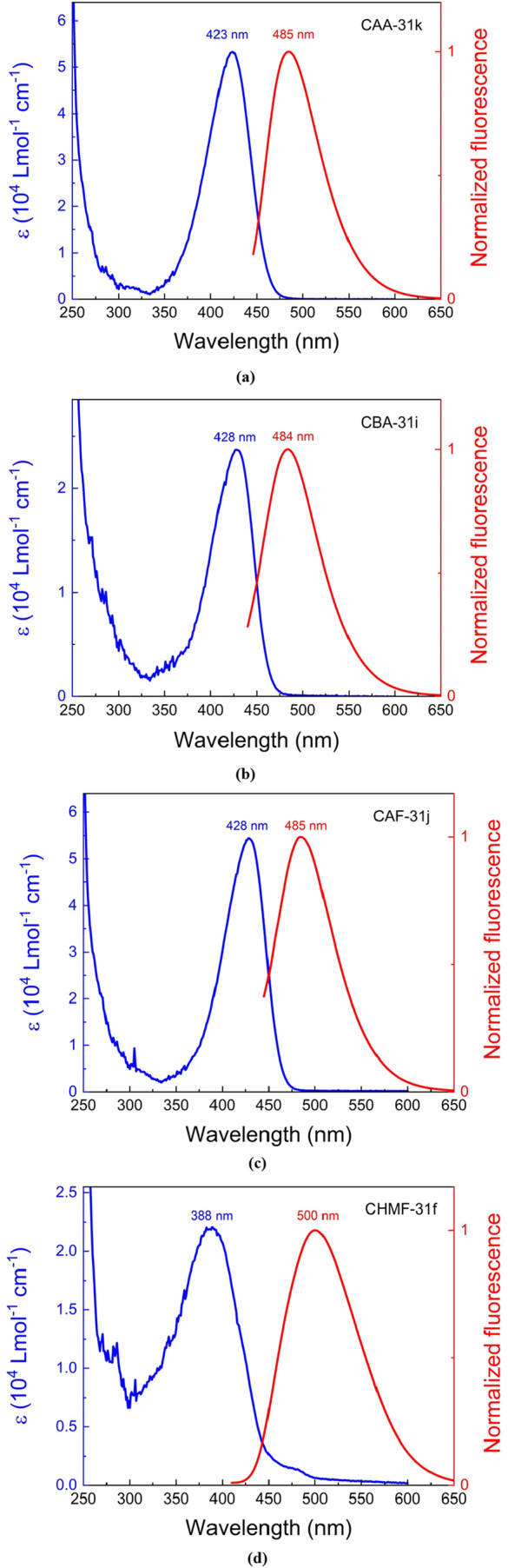
Absorption (blue solid line) and emission (red solid line) spectra of coumarin derivatives (a) CAA-31k, (b) CBA-31i, (c) CAF-31j, and (d) CHMF-31f. CAA-31k, CBA-31i, and CAF-31j exhibit nearly identical absorption maxima at approximately 423 nm (CAA-31k) and 428 nm (CBA-31i and CAF-31j), as well as a common emission maximum at ∼ 485 nm. In contrast, CHMF-31f shows a blue-shifted absorption maximum (∼388 nm) and a red-shifted emission band (∼500 nm). While CAA-31k and CAF-31j display higher molar absorptivities (ε ∼ 5.3 × 10^4^ L mol^–1^ cm^–1^), CBA-31i (ε ∼ 2.4 × 10^4^ L mol^–1^ cm^–1^) and CHMF-31f (ε ∼ 2.2 × 10^4^ L mol^–1^ cm^–1^) exhibit lower ε values.

CAA-31k contains the electron-donating diethylamino group within the coumarin scaffold,^44,45^ which enhance electron density and increase the probability of light absorption,^46^ leading to a higher ε ^47–50^ and a to a narrower electrochemical band gap.^28^ Similarly, **CAF-31j**, which also bears a diethylamino substituent, exhibits a comparably high *ε*. In this case, the combination of the strong electron-donating group with the **benzimidazole moiety** promotes efficient intramolecular charge transfer (ICT) and extended π-conjugation, reinforcing the oscillator strength of the main electronic transition and resulting in absorption and emission characteristics closely matching those of CAA-31k.

Conversely, CBA-31i’s electron-withdrawing bromine substituent reduces electron density in the aromatic ring.^51,52^ Bromine also acts as a potent hydrogen acceptor and may facilitate the formation of reactive radical species with unpaired electrons,^53^ leading to a significantly lower ε despite the similar absorption wavelength.^51,54,55^ In the case of **CHMF-31f**, the absence of a strong electron-donating group and the presence of a **hydroxyl substituent** result in a reduced ICT character. The hydroxyl group promotes hydrogen-bonding interactions with the solvent, increasing excited-state relaxation and contributing to the observed blue-shifted absorption, red-shifted emission, larger Stokes shift, and the lowest ε among the investigated compounds. Overall, while the four derivatives share closely related chromophoric frameworks, variations in donor strength and coupling of thiazole or imidazole groups play a decisive role in modulating their light-absorption efficiency.

The spectral region of the absorption bands observed for the coumarin derivatives investigated in this work is consistent with values previously reported in the literature for coumarin-based fluorophores.^56–58^ For example, the maximum molar absorptivity values obtained here are in good agreement with those reported by Badaro et al.,^59^ namely from approximately 1.6 × 10^4^ M^–1^ cm^–1^ to about 4 × 10^4^ M^–1^ cm^–1^ with absorption maxima in the range of 433–509 nm. More generally, coumarin-based compounds are known to exhibit intense low-energy absorption bands associated with the π-conjugated coumarin framework, and the position and intensity of these bands depend on the substitution pattern and on medium effects.

The differences in ε observed in Figure 2 translate directly into differences in the transition dipole moment between the ground to first excited-state transition dipole moment (μ_01_). A closer inspection of the absorption profiles reveals a non-negligible shoulder in the high-energy region, as shown in Figure 3, indicating that the experimental bands are broader than expected for a single dominant component. To obtain reliable μ_01_values for all four derivatives, the absorption spectra of CAA-31k, CBA-31i, CAF-31j, and CHMF-31f were therefore decomposed into Gaussian sub-bands. At this stage, the spectral decomposition should be regarded as a mathematical fitting procedure that reproduces the experimental spectra and enables quantitative extraction of μ_01_. In other words, the Gaussian spectral decomposition employed in Figure 3 should be regarded as a mathematical procedure used to reproduce the experimental absorption band and to obtain reliable integrated absorption areas required for the transition dipole moment calculation. The fitted components do not represent independent electronic states.

**Figure 3:**
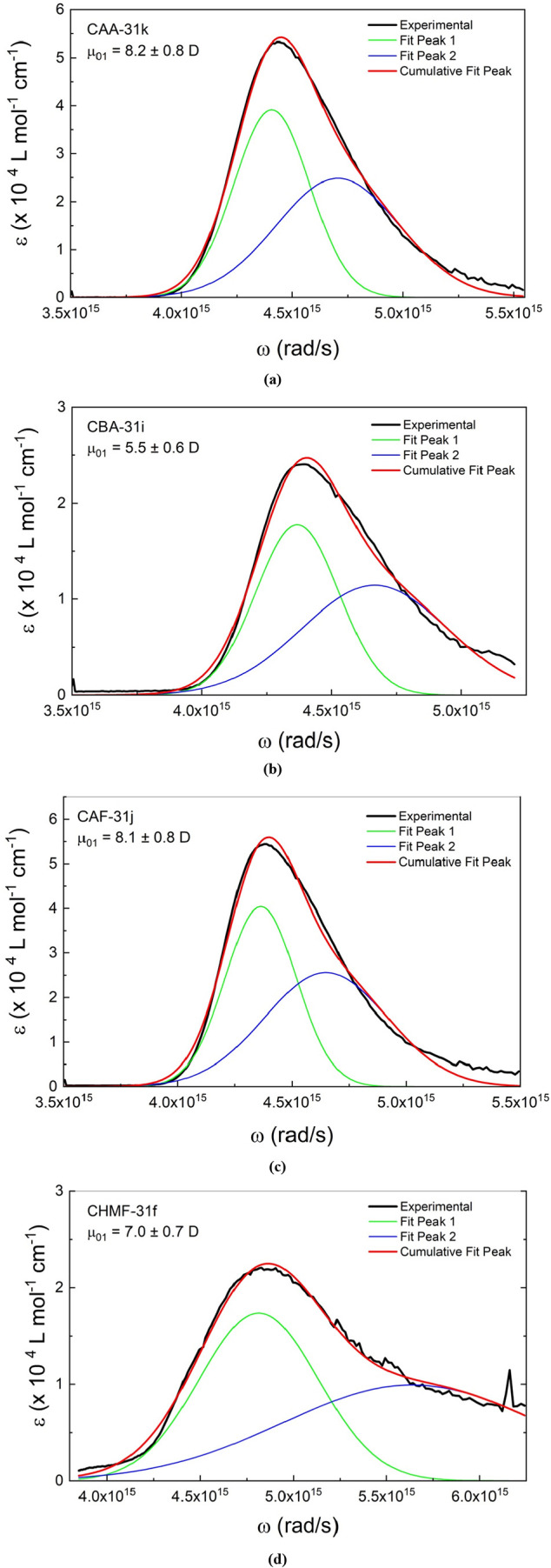
Spectral decomposition of the absorption bands for coumarin derivatives (a) CAA-31k, (b) CBA-31i, (c) CAF-31j, and (d) CHMF-31f. The experimental absorption spectra (black lines) were fitted using the superposition of two Gaussian components (colored lines–green and blue), whose sum (red line) reproduces the measured profiles. This Gaussian decomposition was employed as a mathematical fitting procedure to extract the ground-to-first-excited-state transition dipole moments (μ_01_) for each compound. The resulting μ_01_ values are 8.2 ± 0.8 D (CAA-31k), 5.5 ± 0.6 D (CBA-31i), 8.1 ± 0.8 D (CAF-31j), and 7.0 ± 0.7 D (CHMF-31f).

The highest μ_01_ values are obtained for the diethylamino-substituted derivatives, CAA-31k and CAF-31j with values around 8.0 D, consistent with their ε.values. This enhancement is attributed to the strong electron-donating character of the diethylamino group, which increases electron density and molecular polarizability and strengthens light–matter coupling through more intense charge-transfer-assisted absorption.^28^ In contrast, CBA-31i exhibits a significantly smaller μ_01_ ∼ 5.5 D, in line with its reduced ε, which is consistent with the electron-withdrawing effect of the bromine substituent that decreases electron density within the conjugated system. Finally, CHMF-31f exhibits an intermediate μ_01_ of about 7.0 D despite displaying the lowest molar absorptivity. The μ_01_ values determined here are consistent with the transition dipole moment values previously reported for other coumarin derivatives.^60^

From a mathematical standpoint, this behavior arises from the larger line width of its lowest-energy Gaussian component, which increases the integrated absorption area and therefore contributes significantly to the extracted μ_01_ value. From a chemical perspective, this reflects a distinct electronic balance in CHMF-31f, in which the absence of a strong electron donor and the presence of a hydroxyl group modulate the electronic distribution while still preserving a substantial transition strength within the conjugated chromophore. Additional details regarding the Gaussian fitting protocol and the procedure used to calculate μ_01_ are provided in Section 1 of the Supporting Information.

From a mechanistic point of view, the photophysical behavior of the investigated coumarin-based hybrids may be discussed in terms of a lowest-energy excited state that, in many substituted coumarin systems, is commonly described as being largely dominated by a π → π* transition, although its precise character may vary with the substitution pattern, molecular conformation, and surrounding medium.^61–64^ In this context, the substituents can modulate the redistribution of electron density upon excitation and, consequently, influence the transition dipole moment, the relative stabilization of the excited state, and the spectral positions of the absorption and emission bands.

### 3.2 Fluorescence Anisotropy

Figure 4 presents the fluorescence anisotropy (*r*) measured across the absorption bands of CAA-31k, CBA-31i, CAF-31j, and CHMF-31f, together with their normalized absorbance spectra. For all four compounds, the anisotropy remains quasi-constant over the entire absorption region, despite differences in spectral shape, bandwidth, and substituent effects. In general, significant wavelength-dependent variations in *r* across an absorption band would indicate the involvement of electronic transitions with different transition dipole moment orientations or the presence of distinct photophysical pathways.^65^ The absence of such variations here strongly suggests that the overlapping sub-bands identified through the Gaussian spectral decomposition originate from transitions sharing similar or collinear transition dipole orientations.

**Figure 4:**
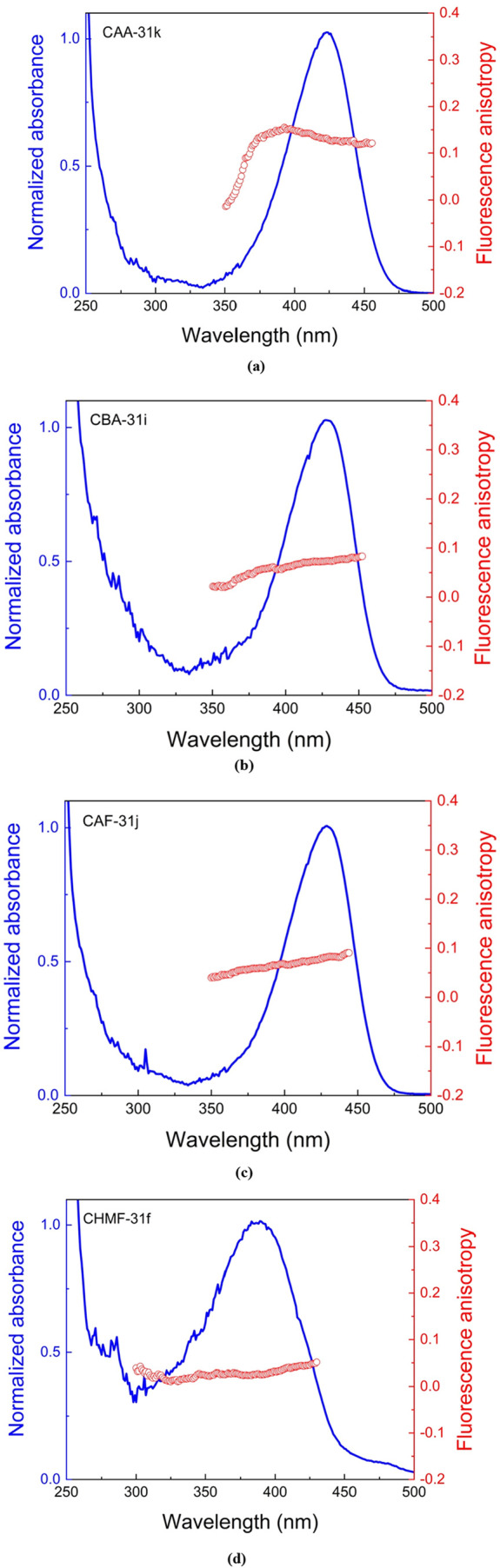
Fluorescence anisotropy (*r*) and normalized absorbance spectra for coumarin derivatives (a) CAA-31k, (b) CBA-31i, (c) CAF-31j, and (d) CHMF-31f. The absorbance spectra (blue lines) exhibit broad bands, while the fluorescence anisotropy (red circles) remains quasi-constant across the absorption range.

This behavior indicates that the absorption bands of all investigated derivatives arise from electronically related π–π* transitions within a common chromophoric framework, rather than from independent or electronically decoupled excited states. Even in the case of CHMF-31f, which exhibits a broader and spectrally shifted absorption profile, the weak dependence of *r* on wavelength implies that the dominant electronic transitions contributing to absorption preserve a consistent dipole orientation. Consequently, the mathematical decomposition of the absorption bands reflects the presence of closely spaced electronic or vibronic components within the same electronic manifold, rather than qualitatively different types of transitions. Irrespective of the specific molecular substituents–bromine in CBA-31i, diethylamino groups in CAA-31k and CAF-31j, or a hydroxyl group in CHMF-31f–the *r* results demonstrate a shared photophysical behavior across the series. In all cases, the most significant vertical electronic transition is located at the maximum of the experimental absorption band, corresponding to the lowest-energy region, and thus provides the most reliable representation of μ_01_. Additional details regarding the determination of *r* are provided in Section 2 of the Supporting Information.

### 3.3 Solvatochromism

The solvatochromism results for CAA-31k, CBA-31i, CAF-31j, and CHMF-31f (Figure 5) demonstrate how solvent polarity modulates their absorption and emission spectra, providing insight into polarity changes upon excitation. For the diethylamino-substituted derivatives CAA-31k and CAF-31j, as well as CBA-31i, the absorption maxima exhibit only minor solvent-dependent shifts, suggesting that the ground-state electronic distribution is comparatively less sensitive to the solvent environment. In contrast, the emission bands display clear red shifts in more polar solvents (e.g., DMSO and ethanol), consistent with stabilization of a more polar excited state and therefore a positive solvatochromic response. The behavior of CHMF-31f follows the same qualitative trend (larger stabilization of the excited state in polar media), but with a noticeably larger absolute Stokes shift across the solvent set, in agreement with its distinct emission behavior and stronger sensitivity to solvation.

**Figure 5:**
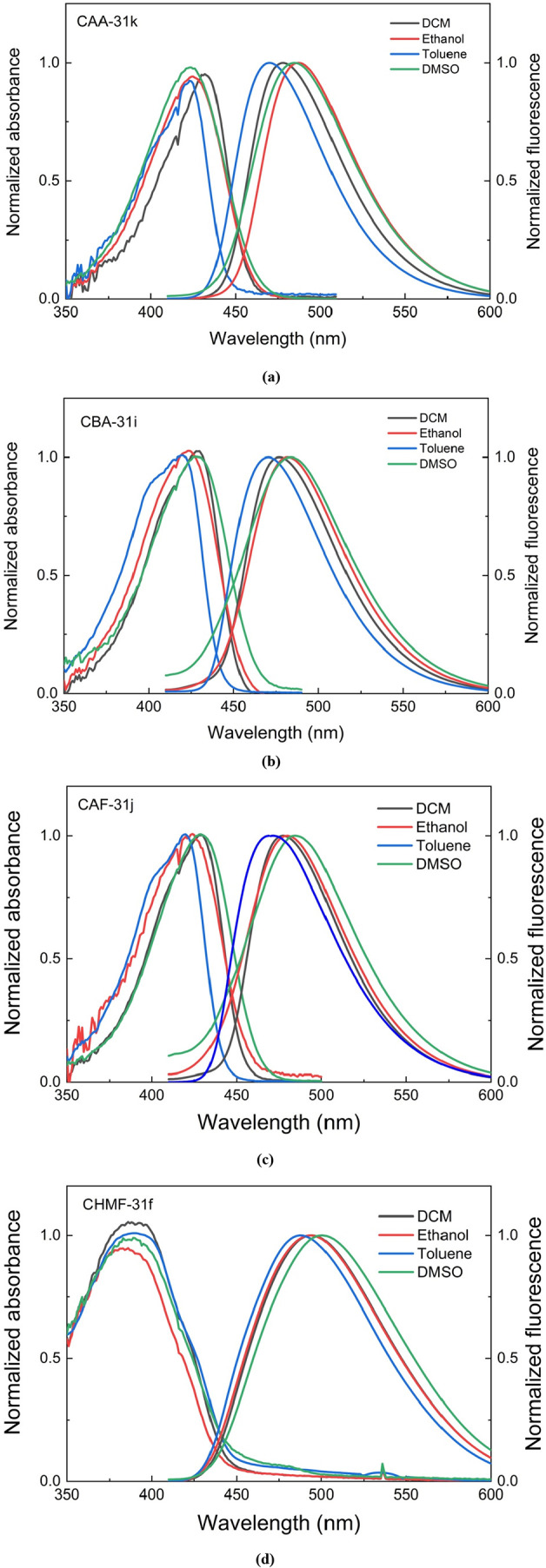
Solvatochromism of (a) CAA-31k, (b) CBA-31i, (c) CAF-31j, and (d) CHMF-31f in solvents of varying polarity (DCM, ethanol, toluene, DMSO). Normalized absorption spectra (left *y*-axis) show minimal solvent dependence, indicating stable ground-state dipole moments. In contrast, normalized emission spectra (right *y*-axis) shift red in polar solvents, reflecting increased excited-state polarity.

To further explore the difference in permanent dipole moments between the ground and first-excited states (Δμ_01_ = μ_11_ – μ_00_), we employed the Lippert-Mataga approach.^66^ Such an approach requires the slope of the solvatochromic shift (Δν) as a function of the Onsager function (*F*), which depends on the solvent’s dielectric constant and refractive index. Specifically, we calculated Δν/Δ*F* in which Δν (in cm^–1^ units) represents the difference between the emission and absorption maxima across different polarity solvents, reflecting the shift in energy levels due to solvent polarity, depicted in Figure 6. The other parameter required to apply the Lippert-Mataga method is the cubic radius of the fluorophore cavity (*a* ^3^), which represents the effective molecular volume interacting with the solvent that can be experimentally estimated by using the Smoluchowski-Einstein diffusion equation.^67^

**Figure 6:**
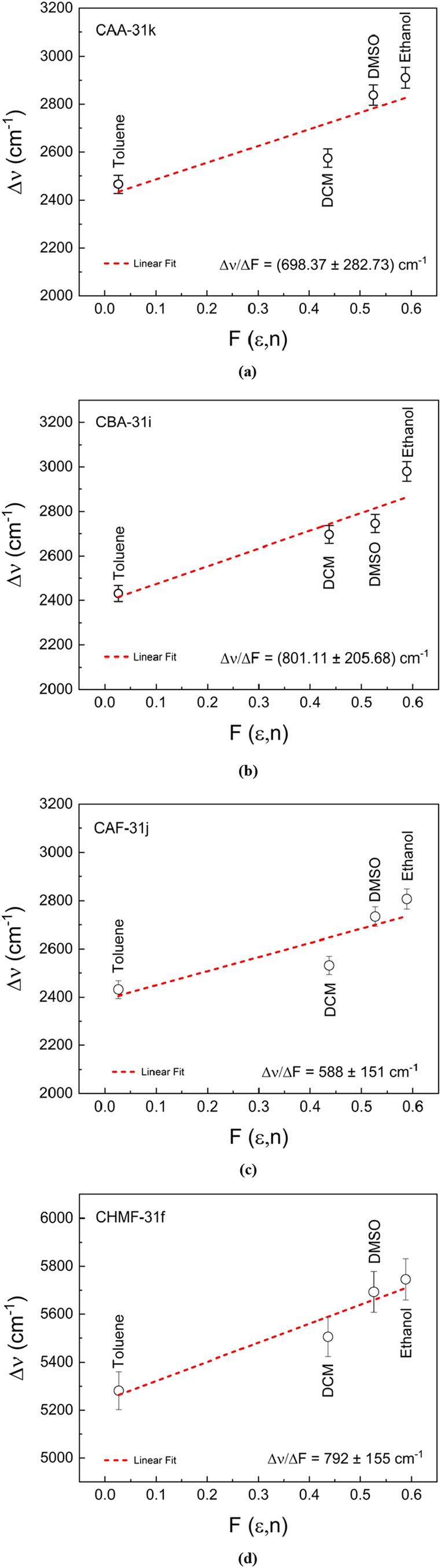
Plots of the solvatochromic shift (Δν) versus the Onsager function (F) for (a) CAA-31k, (b) CBA-31i, (c) CAF-31j, and (d) CHMF-31f, used to determine the slope Δν/Δ*F* for each compound. The resulting Δν/Δ*F* values are 698 ± 283 cm^–1^ (CAA-31k), 801 ± 206 cm^–1^ (CBA-31i), 588 ± 151 cm^–1^ (CAF-31j), and 792 ± 155 cm^–1^ (CHMF-31f). The associated uncertainties reflect experimental variability and deviations from ideal dielectric continuum behavior, particularly due to solvent-specific interactions.

Figure 6 confirms that all four derivatives exhibit an overall increase in Δν with increasing *F*, consistent with excited-state stabilization in polar solvents. The extracted slopes Δν/Δ*F* are 698 ± 283 cm^–1^ for CAA-31k, 801 ± 206 cm^–1^ for CBA-31i, 588 ± 151 cm^–1^ for CAF-31j, and 792 ± 155 cm^–1^ for CHMF-31f. Within the Lippert–Mataga framework, these slopes reflect the magnitude of polarity change upon excitation, i.e., they are proportional to (Δμ_01_)^2^/*a* ^3^. Accordingly, CBA-31i and CHMF-31f exhibit the steepest slopes, suggesting a larger solvent-dependent stabilization of the excited state relative to CAA-31k and especially CAF-31j. Chemically, this trend is consistent with the stronger acceptor/less-donor balance in CBA-31i (electron-withdrawing bromine) and the distinct electronic distribution in CHMF-31f (hydroxyl-containing scaffold with enhanced specific solvation), whereas the diethylamino-bearing derivatives CAA-31k and CAF-31j show slightly smaller slopes, indicating a comparatively reduced solvatochromic sensitivity under the same solvent set. Further information on the solvatochromic analysis using the Lippert–Mataga equation is provided in the Supporting Information (Section 3).

The high uncertainty in the Δν/Δ*F* values arises from the inherent variability in solvatochromic measurements. This uncertainty stems from factors such as the complex, nonequilibrium nature of solvent–solute interactions during fluorescence and slight inconsistencies in solvent polarity parameters, such as dielectric constant and refractive index.^68^ These variations, along with impurities or deviations in solvent behavior, impact the precision of the Onsager function and contribute to the observed standard deviations in the Δν/Δ*F* slopes.

### 3.4 Fluorescence Lifetime and Quantum Yield

The fluorescence lifetimes (τ_FL_) of CAA-31k, CBA-31i, and CAF-31j were measured but found to be equal to or shorter than 0.7 ns, which is below the temporal resolution of the photodetector employed. As a result, the fluorescence decay of these compounds could not be experimentally resolved. In contrast, CHMF-31f exhibits a measurable fluorescence lifetime of approximately 3.0 ns, allowing direct lifetime determination. The lack of resolvable lifetime data for most compounds posed a limitation, as accurate τ_FL_ values are required to estimate the fluorophore cavity size via the Smoluchowski–Einstein diffusion equation.^67^ Consequently, an alternative strategy was required to determine the cavity parameter *a* ^3^ for the solvatochromic analysis and subsequent estimation of Δμ_01_.

To overcome this limitation, quantum-chemical calculations (QCC) were performed using Gaussian 16 software package^69^ to theoretically estimate the cavity size parameter *a* ^3^ for all four compounds. The ground-state geometries were optimized at the CAM-B3LYP/6–311+G­(d,p)^70^ level of theory using the OPT keyword. The *a* ^3^ value was estimated from the optimized geometry using the VOLUME keyword,^71^ which computes the molecular volume as the space enclosed by an electron-density contour; in the default implementation, this contour corresponds to 0.001 electrons/Bohr.^3^ Solvent effects were included through the integral equation formalism polarizable continuum model (IEF-PCM),^72^ in order to account for solute–solvent electrostatic interactions within a continuum description of the medium. The resulting theoretical molecular volumes were then used as estimates of the effective cavity size parameter *a* ^3^ in the Lippert–Mataga formalism. Further details, as well as the optimized Cartesian coordinates of the investigated compounds, are provided in Section 4 of the SM.

The theoretically obtained *a* ^3^values were then incorporated into the Lippert–Mataga formalism, together with the experimentally determined Δν/Δ*F* slopes derived from the solvatochromic measurements. Details of the computational protocol used to estimate *a* ^3^ are provided in the Supporting Information (Section 4). Using this semiempirical approach, which combines experimental solvatochromic data with theoretically derived cavity parameters, the dipole moment differences upon excitation (Δμ_01_) were estimated for CAA-31k, CBA-31i, CAF-31j, and CHMF-31f, as summarized in Table 1. Although this method relies on theoretical assumptions for the cavity term, it enables a consistent and comparative evaluation of excited-state polarity changes across the entire series.

**Table 1:** Spectroscopic Parameters for All Investigated Compounds, Including Transition Dipole Moment (μ_01_), Solvatochromic Slope (Δν/Δ*F*), Fluorophore Cavity Volume (*a* ^3^), Fluorescence Quantum Yield (ϕ_FL_), and Dipole Moment Difference (Δμ_01_)­^a^

	μ_01_ (D)	Δν/Δ*F* (cm^–1^)	*a* ^3^ (Ȧ^3^)	ϕ_FL_ (%)	Δμ_01_ (D)
**CAA-31k**	5.5 ± 0.6	811 ± 205	192.1	9.1 ± 0.9	5.1 ± 0.8
**CBA-31i**	8.2 ± 0.8	698 ± 282	133.4	5.8 ± 0.6	4.6 ± 0.7
**CAF-31j**	8.1 ± 0.8	588 ± 282	171.9	5.7 ± 0.6	4.5 ± 0.7
**CHMF-31f**	7.0 ± 0.7	792 ± 155	127.3	48.4 ± 4.8	4.5 ± 0.7

a The μ_01_ and ϕ_FL_ values were determined in DMSO, with the fluorescence quantum yield calculated using Coumarin 480 as an external reference. The molecular volume (*a* ^3^) was theoretically estimated in the DMSO medium.

To complete the characterization of the linear photophysical properties, the fluorescence quantum yields (ϕ_FL_) of all compounds were determined using Coumarin 480 in DMSO as an external reference. The ϕ_FL_ is listed in Table 1 therefore correspond to values obtained under identical reference conditions. The results reveal marked differences in radiative efficiency across the series. While CAA-31k, CBA-31i, and CAF-31j exhibit relatively modest quantum yields in DMSO (ϕ_FL_ ≈ 5.0–9.0%), CHMF-31f displays a substantially higher fluorescence quantum yield (ϕ_FL_ = 48.4 ± 4.8%), consistent with its longer fluorescence lifetime and reduced nonradiative decay pathways. These trends highlight the strong influence of molecular structure on excited-state relaxation dynamics, with the hydroxyl-substituted CHMF-31f favoring efficient radiative decay compared to the other derivatives.

Finally, it should be noted that the ϕ_FL_ of the coumarins studied in this work fall within the broad range reported in the literature for coumarin-based dyes and fluorophores, whose emission efficiency is known to depend strongly on molecular structure and medium effects.^59,73–76^ For example, Badaro et al.^59^ reported coumarin analogues with fluorescence quantum yields reaching up to 92% in organic media, while other studies have shown that coumarin derivatives can also display substantially lower values depending on substitution pattern, conformational flexibility, and solvent environment. In particular, solvent-dependent studies on Coumarin 102 and Coumarin 153 ^73^ have shown that their fluorescence quantum yields vary significantly across different media, with reported values of 43–62% and 21–38%, respectively, under the conditions examined.

### 3.5 Multiphoton Excitation

In the final stage of our optical characterization, we investigated the multiphoton excitation behavior of all compounds using femtosecond pulses to assess their potential as multiphoton photoluminescent molecules. This experiment aimed to determine if these compounds’ fluorescence emission could be effectively triggered via two-photon (800 nm excitation) and three-photon (1200 nm) excitation processes. The ability of these molecules to undergo multiphoton excitation is essential for applications in deep-tissue imaging and high-resolution fluorescence microscopy,^77^ where longer wavelength excitation offers the advantages of deeper penetration and reduced photodamage to biological samples.

The fluorescence intensity for both compounds was measured as a function of the laser pumping power at 800 nm for two-photon excitation and 1200 nm for three-photon excitation. The fluorescence intensity was plotted against each experiment’s increasing laser pump power on a log–log scale, as shown in Figures 7 and 8. The slope of this plot provides evidence of the nature of the excitation process. In the case of two-photon excitation (Figure 7), a slope of 2 confirms the two-photon absorption process.^78^ In contrast, a slope around 3 in the three-photon excitation experiment (Figure 8) indicates successful three-photon absorption.^79^ The inset spectra depicted in Figures 7 and 8 illustrate that the multiphoton excitation wavelengths access the same fluorescence emission bands as one-photon excitation, confirming the integrity and consistency of the photoluminescence. These results point out the versatility of all compounds as potential multiphoton fluorescence applications. The successful demonstration of multiphoton-triggered fluorescence suggests that these coumarin derivatives are promising candidates for advanced photonic applications requiring nonlinear optical properties.^66,80^

**Figure 7:**
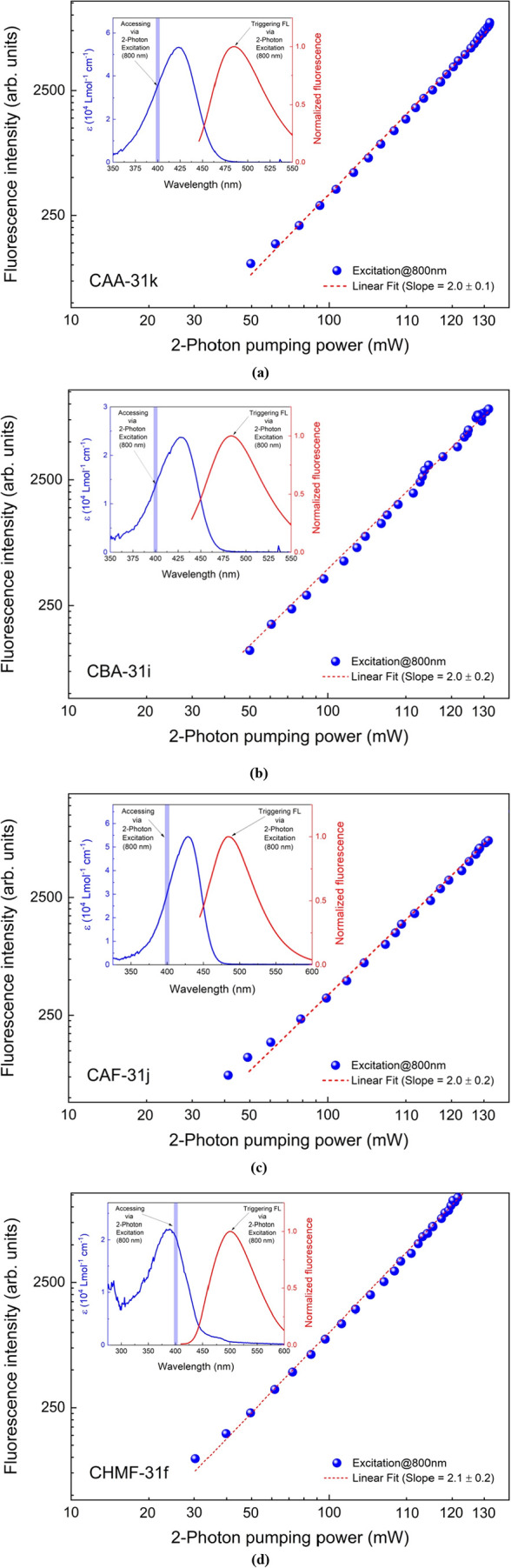
Two-photon excitation fluorescence intensity plotted against laser power for (a) CAA-31k, (b) CBA-31i, (c) CAF-31j, and (d) CHMF-31f using 800 nm excitation. The linear fit (red dashed line) confirms two-photon absorption with a slope of approximately 2.0. Insets in each plot show the absorbance and emission spectra, highlighting the two-photon excitation pathways.

**Figure 8:**
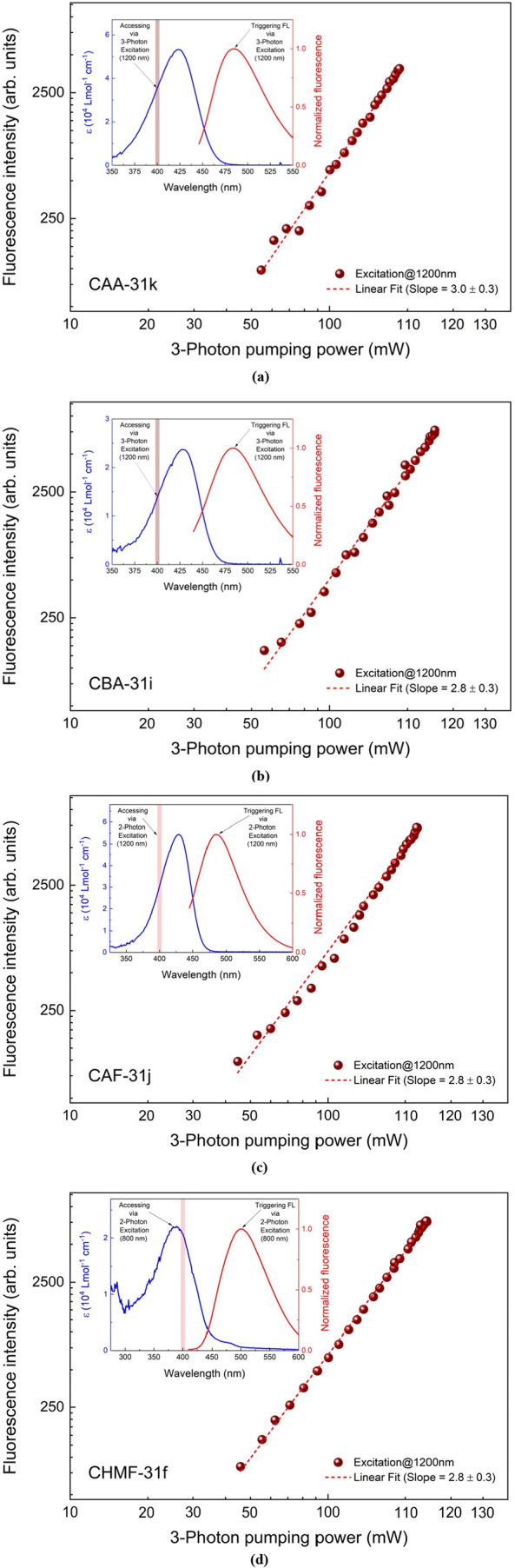
Three-photon excitation fluorescence intensity plotted against laser power for (a) CAA-31k, (b) CBA-31i, (c) CAF-31j, and (d) CHMF-31f using 1200 nm excitation. The linear fit (red dashed line) confirms three-photon absorption, with slopes of approximately 2.8 and 3.0, respectively. Insets in each plot show the absorbance and emission spectra, highlighting the three-photon excitation pathways.

## 4 Conclusions

This work investigated the linear optical properties and multiphoton excitation response of four coumarin-based derivatives: CAA-31k, CBA-31i, CAF-31j, and CHMF-31f. By systematically comparing compounds bearing electron-donating and electron-withdrawing substituents as well as coupling the coumarin nuclei with different heteroaromatic moieties, acceptor units like benzothiazole or benzimidazole, this study elucidates how subtle structural variations govern their photophysical behavior.

UV–vis absorption and fluorescence measurements showed that CAA-31k, CBA-31i, and CAF-31j exhibit nearly identical absorption and emission maxima, while differing markedly in molar absorptivity and ground-to-excited-state transition dipole moments. The enhanced molar absorptivity and larger μ_01_ values observed for CAA-31k and CAF-31j are attributed to the presence of strong diethylamino donor groups, which favor intramolecular charge transfer and strengthen light–matter interaction. In contrast, CHMF-31f displays a distinct spectral profile characterized by a larger Stokes shift and lower molar absorptivity, reflecting a different electronic balance associated with its hydroxyl-substituted framework.

Solvatochromic analyses, interpreted within the Lippert-Mataga formalism, revealed a positive solvatochromic response for all compounds, indicating stabilization of more polar excited states in polar solvents. While fluorescence lifetime measurements for CAA-31k, CBA-31i, and CAF-31j were limited by instrumental resolution (lifetimes below 0.7 ns), CHMF-31f exhibited a measurable lifetime of approximately 3.0 ns. To ensure a consistent analysis across the full series, quantum-chemical calculations were employed to estimate the fluorophore cavity size, enabling a semiempirical determination of permanent transition dipole moments for all derivatives.

Multiphoton excitation experiments demonstrated that all four compounds are active under two- and three-photon excitation, producing fluorescence under near-infrared irradiation. The power-law dependence of the emission intensity confirmed the nonlinear nature of these processes. From a practical perspective, these results are especially relevant because they report that the investigated dyes are capable of producing measurable fluorescence under localized multiphoton excitation, which is essential for applications such as two- and three-photon microscopy, even though multiphoton absorption cross sections were not determined in the present study. Within this context, differences in fluorescence efficiency become particularly important for assessing the practical suitability of each compound under nonlinear optical excitation conditions.

CHMF-31f clearly emerges as the most promising photoluminescent probe among the investigated compounds, primarily due to its high fluorescence quantum yield of approximately 48%, which is about four times higher than that of the other derivatives. Combined with its longer excited-state lifetime, which facilitates efficient signal detection using conventional fast photodetectors, CHMF-31f is particularly attractive for biological applications. Consequently, its high radiative efficiency, together with robust emission under one-, two-, and three-photon excitation, makes CHMF-31f especially well-suited for applications requiring bright fluorescence under both linear and nonlinear optical excitation regimes.

## Supplementary Material



## Notes

CAA-31k and CBA-31i optimized geometries obtained using the CAM-B3LYP/6-311+(d,p) level of theory in DMSO medium, and CAF-31j and CHMF-31d optimized geometries obtained using the CAM-B3LYP/6-311+(d,p) level of theory in DMSO medium (PDF)
