# Supplementary material for: Coumarin-Based Hybrids: From Linear Photophysical Properties to Multiphoton Excitation Behavior
Source: ACS Phys Chem Au. 2026 May 14;6(4):726–38. doi: 10.1021/acsphyschemau.6c00010 (PMC13397450; doi:10.1021/acsphyschemau.6c00010)
Supplement: Supplementary file 1 [file pg6c00010_si_001.pdf]

Supplementary Material

Coumarin-Based Hybrids: From Linear Photophysical Properties to Multiphoton Excitation Behavior

Luis M. G. Abegão[a,*], Leonardo R. de Almeida[b], Juliana G. M. Lima[b], Luciana M. Ramos[b], Hamilton B. Napolitano[b], João V. Valverde[c], Cleber R. Mendonça[c], Leonardo De Boni[c] and Leandro H. Z. Cocca[d,*]

[a] Sensors, Instrumentation and Applied Photonics Group, Department of Physics, Federal University of Sergipe, São Cristóvão, SE, Brazil.

[b] Laboratory of Medicinal Chemistry and Organic Synthesis, State University of Goiás, Anápolis, GO, Brazil.

[c] Photonics Group, Institute of Physics of São Carlos, University of São Paulo, São Carlos, SP, Brazil.

[d] Photonics Group, Institute of Physics, Federal University of Goiás, Goiânia, GO, Brazil.

* Authors to whom correspondence should be addressed; E-Mail: luis.abegao@academico.ufs.br and leandro.zucolottococca@gmail.com

Section 1: Details on the calculation of the transition dipole moment ($\boldsymbol{\mu_{01}}$)

Equation 1 was used to determine the electronic transition dipole moment (μ_{01}) [1], in which $L = \frac{3n^2}{2n^2+1}$ is the Onsager local field factor, h is the Planck constant, n is the refractive index of the used solvents, N_A is the Avogadro number, ω_{01} is the frequency transition (in rad/s), and ε is the molar absorptivity.

$$\mu_{01} = \sqrt{\frac{3 \text{ x } 10^3 \ln(10)}{8\pi^3} \frac{hc}{N_A} \frac{n}{L^2} \frac{1}{\omega_{01}} \int \varepsilon(\omega) d\omega} \quad (1)$$

Section 2: Details on the calculation of the fluorescence anisotropy ($\boldsymbol{r}$)

The fluorescence anisotropy (r) was determined by using **Equation 2**, in which $I_{\parallel}$ and $I_{\perp}$ are the fluorescence emission intensities collected parallel and perpendicular to the excitation light polarization direction, respectively [1].

$$r = \frac{\frac{I_{\parallel}}{I_{\perp}} - 1}{\frac{I_{\parallel}}{I_{\perp}} + 2} \quad (2)$$

Section 3: Details on the calculation of the solvatochromic parameters

Equation 3 represents the Lippert-Mataga equation [1], where the experimentally obtained parameter is the Stokes shift ($\Delta\nu$). The Onsager polarity function is described by $\Delta F = \frac{\varepsilon-1}{2\varepsilon+1} - \frac{n^2-1}{2n^2+1}$. In this equation ε and n are the dielectric constant and refractive index of the solvent used, respectively. Therefore, the difference between the first excited state permanent electric dipole moment and the ground state permanent electric dipole moment ($\Delta\mu_{01} = \mu_{11} - \mu_{00}$) can be isolated and determined [2], once c represents the speed of light, h is Planck's constant, and a^3 is the cubic radius of the molecule as surrounded by the solvent. It is

important to highlight that a^3, in this particular case, was determined theoretically as outlined in Section 5.

$$\Delta\nu = \frac{2}{hca^3}|\Delta\mu_{01}|^2\Delta F \tag{3}$$

Section 4: Details of the QCC performed using Gaussian 16 to theoretically estimate a^3

The Gaussian 16 program [3] package was employed to estimate the a^3 of the studied molecules in a DMSO medium. The CAM-B3LYP/6-311+G(d,p) [4] level of theory was initially used to optimize the molecular geometry, followed by a volume calculation [5]. Both procedures were performed in solvent medium using the polarizable continuum model (PCM) [6] with the integral equation formalism variant (IEF-PCM) [7] The optimized geometries in Cartesian coordinates are provided in **Table S1**. The a^3 value was obtained from the optimized geometry using the **Volume** keyword, which calculates the molecular volume defined as the space enclosed by a 0.001 electrons/$Bohr^3$ density contour. The resulting a^3 value was subsequently used as an input parameter in **Equation 3.**

Table S1: CAA-31k and CBA-31i optimized geometries obtained using the CAM-B3LYP/6-311+(d,p) level of theory in DMSO medium.

**CAA-31k in DMSO**	**CBA-31i in DMSO**
**CAM-B3LYP/6-311+G(d,p)**	**CAM-B3LYP/6-311+G(d,p)**
O 2.85327 -2.19482 1.91709	Br -13.07182 5.72866 3.71662
C 3.83305 -2.09964 1.18542	C -11.64422 5.69793 2.47676
O 4.63125 -0.95678 1.22811	C -11.23793 6.88529 1.86398
C 5.71505 -0.77074 0.35014	C -10.18580 6.86426 0.94947
C 6.43227 0.42575 0.45482	C -9.55965 5.65267 0.66629
C 7.52367 0.70414 -0.38495	O -8.50918 5.66656 -0.26695
N 8.24719 1.88893 -0.29610	C -7.84030 4.50293 -0.64784
C 7.98814 2.88607 0.75257	C -8.23226 3.24879 0.05114
C 6.89781 3.85828 0.32503	C -7.57969 2.00721 -0.23084
C 9.30803 2.23417 -1.25235	N -8.18844 0.83763 -0.33729
C 10.64982 1.67286 -0.80394	C -7.28032 -0.17967 -0.57523
C 7.83608 -0.29996 -1.32395	C -5.94418 0.21168 -0.60894
C 7.11312 -1.50147 -1.42631	C -4.90623 -0.70770 -0.83514
C 6.03926 -1.74057 -0.58028	C -5.24289 -2.05082 -1.03050
C 5.25813 -2.96276 -0.62937	C -6.57075 -2.46462 -0.99947
C 4.22971 -3.15537 0.21450	C -7.59504 -1.53959 -0.77205
C 3.49966 -4.38091 0.11551	S -5.85253 1.90295 -0.32962
N 3.17284 -4.98169 -1.01671	C -9.24610 3.24119 0.93435
C 2.49992 -6.16734 -0.77741	O -6.98518 4.54446 -1.52565
C 2.34876 -6.50522 0.56524	C -9.95420 4.46441 1.27062
C 1.69354 -7.68161 0.96603	C -11.00859 4.48744 2.18614
C 1.18301 -8.52537 -0.02541	H -11.73383 7.82575 2.09103
C 1.32279 -8.20815 -1.37272	H -9.86051 7.77942 0.46271
C 1.97924 -7.03493 -1.75896	H -3.86962 -0.38521 -0.85945
S 3.07820 -5.28778 1.53112	H -4.45490 -2.77967 -1.20931
H 6.09984 1.13446 1.20540	H -6.81717 -3.51261 -1.15355
H 8.90343 3.45858 0.95083	H -8.63246 -1.85796 -0.74789
H 7.74171 2.41558 1.71089	H -9.55695 2.32333 1.42778
H 7.17725 4.38272 -0.59475	H -11.33610 3.57164 2.67215
H 6.73268 4.60753 1.10554	
H 5.94818 3.34756 0.13928	
H 9.06436 1.90590 -2.26886	
H 9.39785 3.32561 -1.32645	
H 10.63142 0.58087 -0.73761	
H 10.92795 2.05999 0.18195	
H 11.43390 1.95640 -1.51296	
H 8.67053 -0.18158 -2.00893	
H 7.41056 -2.23360 -2.17304	
H 5.53814 -3.71558 -1.36177	
H 1.58390 -7.92935 2.01755	
H 0.66966 -9.44052 0.26274	
H 0.91958 -8.87358 -2.13253	
H 2.08992 -6.78280 -2.80888	

Table S2: CAF-31j and CHMF-31d optimized geometries obtained using the CAM-B3LYP/6-311+(d,p) level of theory in DMSO medium.

CAF-31j in DMSO	CHMF-31f in DMSO
**CAM-B3LYP/6-311+G(d,p)**	**CAM-B3LYP/6-311+G(d,p)**
O 0.33970 2.50097 0.00013	O -1.34340 2.52144 0.08872
C 0.98892 1.47724 0.00008	C -0.72152 1.47717 0.10982
O 2.34709 1.61208 0.00017	O 0.63928 1.58044 0.16679
C 3.20814 0.55643 0.00006	C 1.47921 0.50348 0.19851
C 4.56248 0.84370 0.00005	C 2.82929 0.76437 0.25421
C 5.45573 -0.21669 -0.00006	C 3.74853 -0.30379 0.29571
O 6.79400 -0.03494 -0.00008	N 5.08756 -0.08331 0.38801
C 5.00037 -1.54414 -0.00016	C 5.62719 1.27535 0.41481
C 3.65284 -1.80109 -0.00016	C 5.75425 1.93285 -0.95690
C 2.71947 -0.75011 -0.00005	C 6.04336 -1.19078 0.40332
C 1.30817 -0.93106 -0.00004	C 6.37598 -1.75800 -0.97382
C 0.45515 0.12324 0.00006	C 3.21923 -1.62859 0.25029
C -0.99413 -0.08427 0.00008	C 1.87199 -1.85338 0.19476
N -1.57125 -1.26334 0.00025	C 0.94934 -0.79079 0.16891
C -2.92726 -1.00181 0.00019	C -0.45367 -0.93648 0.10819
C -3.15075 0.38324 -0.00003	C -1.28597 0.14264 0.07987
C -4.42927 0.93799 -0.00021	C -2.73593 -0.03145 0.01937
C -5.50879 0.07032 -0.00014	N -3.34480 -1.19606 -0.00843
C -6.92047 0.59566 -0.00017	C -4.69250 -0.89992 -0.06373
C -5.28663 -1.32512 0.00004	C -4.87866 0.49398 -0.06836
C -4.02003 -1.87299 0.00022	C -6.13986 1.07924 -0.11938
N -1.89500 0.94263 -0.00013	C -7.22510 0.22120 -0.16638
H 4.89673 1.87364 0.00013	C -7.05722 -1.17416 -0.16255
H 7.01741 0.90290 0.00006	C -5.80013 -1.74908 -0.11171
H 5.72714 -2.34557 -0.00025	N -3.60819 1.01848 -0.01480
H 3.29295 -2.82266 -0.00024	H 3.14455 1.79576 0.26045
H 0.90027 -1.93491 -0.00011	H 5.01168 1.88926 1.07537
H -4.57639 2.01151 -0.00039	H 6.60866 1.21952 0.88508
H -7.46883 0.24984 0.87965	H 4.79254 1.98251 -1.47006
H -7.46929 0.24879 -0.87928	H 6.45001 1.38319 -1.59231
H -6.93803 1.68593 -0.00082	H 6.13081 2.95185 -0.84437
H -6.14792 -1.98428 -0.00001	H 5.66916 -1.97850 1.05941
H -3.87229 -2.94628 0.00036	H 6.95284 -0.81721 0.87320
H -1.62806 1.91812 -0.00019	H 5.48376 -2.12613 -1.48308
	H 7.07510 -2.59086 -0.87113
	H 6.84103 -1.00198 -1.60796
	H 3.88335 -2.47843 0.25647
	H 1.49885 -2.87035 0.16526
	H -0.88668 -1.92981 0.08398
	H -6.26803 2.15445 -0.12240
	H -8.22606 0.63333 -0.20698
	H -7.93392 -1.80950 -0.20043
	H -5.67133 -2.82473 -0.10882
	H -3.31223 1.98580 0.00109

References

[1] Lakowicz, J. R. (Ed.). (2006). *Principles of fluorescence spectroscopy*. Boston, MA: springer US.

[2] Abegão, L. M., Fonseca, R. D., Ramos, T. N., Mahuteau-Betzer, F., Piguel, S., Joatan R Jr, J., ... & De Boni, L. (2018). Oxazole dyes with potential for photoluminescence bioprobes: A two-photon absorption study. *The Journal of Physical Chemistry C*, *122*(19), 10526-10534.

[3] Frisch, M. J. (2016). Gaussian 16, Revision B. 01/Gaussian.

[4] Yanai, T., Tew, D. P., & Handy, N. C. (2004). A new hybrid exchange–correlation functional using the Coulomb-attenuating method (CAM-B3LYP). *Chemical physics letters*, *393*(1-3), 51-57.

[5] Abegão, L. M., Cocca, L. H., Mulatier, J. C., Pitrat, D., Andraud, C., Misoguti, L., ... & De Boni, L. (2021). Effective π-electron number and symmetry perturbation effect on the two-photon absorption of oligofluorenes. *Physical Chemistry Chemical Physics*, *23*(34), 18602-18609.

[6] Mennucci, B. (2012). Polarizable continuum model. *Wiley Interdisciplinary Reviews: Computational Molecular Science*, *2*(3), 386-404.

[7] Tomasi, J., Mennucci, B., & Cancès, E. (1999). The IEF version of the PCM solvation method: an overview of a new method addressed to study molecular solutes at the QM ab initio level. *Journal of Molecular Structure: THEOCHEM*, *464*(1-3), 211-226.
